# Spontaneous Intracerebral Hemorrhage (ICH) associated with pregnancy and SARS-CoV-2 infection: a case report

**DOI:** 10.1186/s12884-021-04345-9

**Published:** 2022-01-06

**Authors:** Parisa Dini, Soheila Aminimoghaddam, Zahra Mirzaasgari, Neda Rahimian, Samaneh Tanhapour Khotbehsara, Meysam Abolmaali

**Affiliations:** 1 Department of Gynecology, Firoozgar Hospital, Iran University of Medical Sciences, Tehran, Iran; 2 Shefa Neuroscience Research Center, Khatam Alanbia Hospital, Tehran, Iran; 3 Department of Neurology, Firoozgar Hospital, Iran University of Medical Sciences, Tehran, Iran; 4 Department of Internal Medicine, Firoozgar Hospital, Iran University of Medical Sciences, Tehran, Iran; 5 School of Medicine, Iran University of Medical Sciences, Tehran, Iran

**Keywords:** COVID-19, Intracerebral hemorrhage, Pregnancy, Case report

## Abstract

**Background:**

Coronavirus Disease 2019 (COVID-19) is predominately known as a respiratory disease associated with pneumonia, acute respiratory distress syndrome and multiorgan failure. However, extra-pulmonary complications of severe acute respiratory syndrome coronavirus 2 (SARS-CoV-2) are increasingly being recognized. In this regard, some studies implied the hemostatic and vascular involvements in patients with SARS-CoV-2 infection.

**Case presentation:**

We describe a case of spontaneous Intracerebral Hemorrhage (ICH) in a pregnant patient with COVID-19 and history of cesarean section a week before the occurrence of ICH. The patient underwent emergent craniotomy with acceptable outcome. Hemorrhagic events, including ICH, may happen during COVID-19 infection with several possible mechanisms.

**Conclusion:**

COVID-19 patients, especially high-risk groups, are at a risk of intracranial hemorrhage. Therefore, close follow-up must be maintained and hemorrhagic events must be kept in mind in these cases.

## Background

On January 2020, the World Health Organization (WHO) was informed of 44 patients with pneumonia of unknown etiology, by the national authorities of China. These cases later confirmed to be caused by a novel type of coronavirus; which was then named 2019 novel coronavirus (2019-nCoV) and on March 2020, WHO characterized 2019-nCoV outbreak as a pandemic [1, 2]. Soon after the beginning of the pandemic, physicians witnessed several extrapulmonary complications of coronavirus disease 2019 (COVID-19) that were affecting the patients and considered these complications to be the main cause of death. The coagulation system imbalances were perceived as one of the most important complications of COVID-19 [3, 4]. So far, some studies have implied both thrombotic and hemorrhagic events in the COVID-19 patients [5–7]. Also pregnancy has an effect on hemostasis by causing a hyper-coagulopathy state [8]. It has been seen that pregnant women are more likely to develop severe COVID-19 [9]. However, there were no reports of hemostasis disorders in pregnant women with COVID-19. Here we report a case of intracranial hemorrhage in a COVID-19 patient after her childbirth.

## Case presentation

**Fig. 1:**
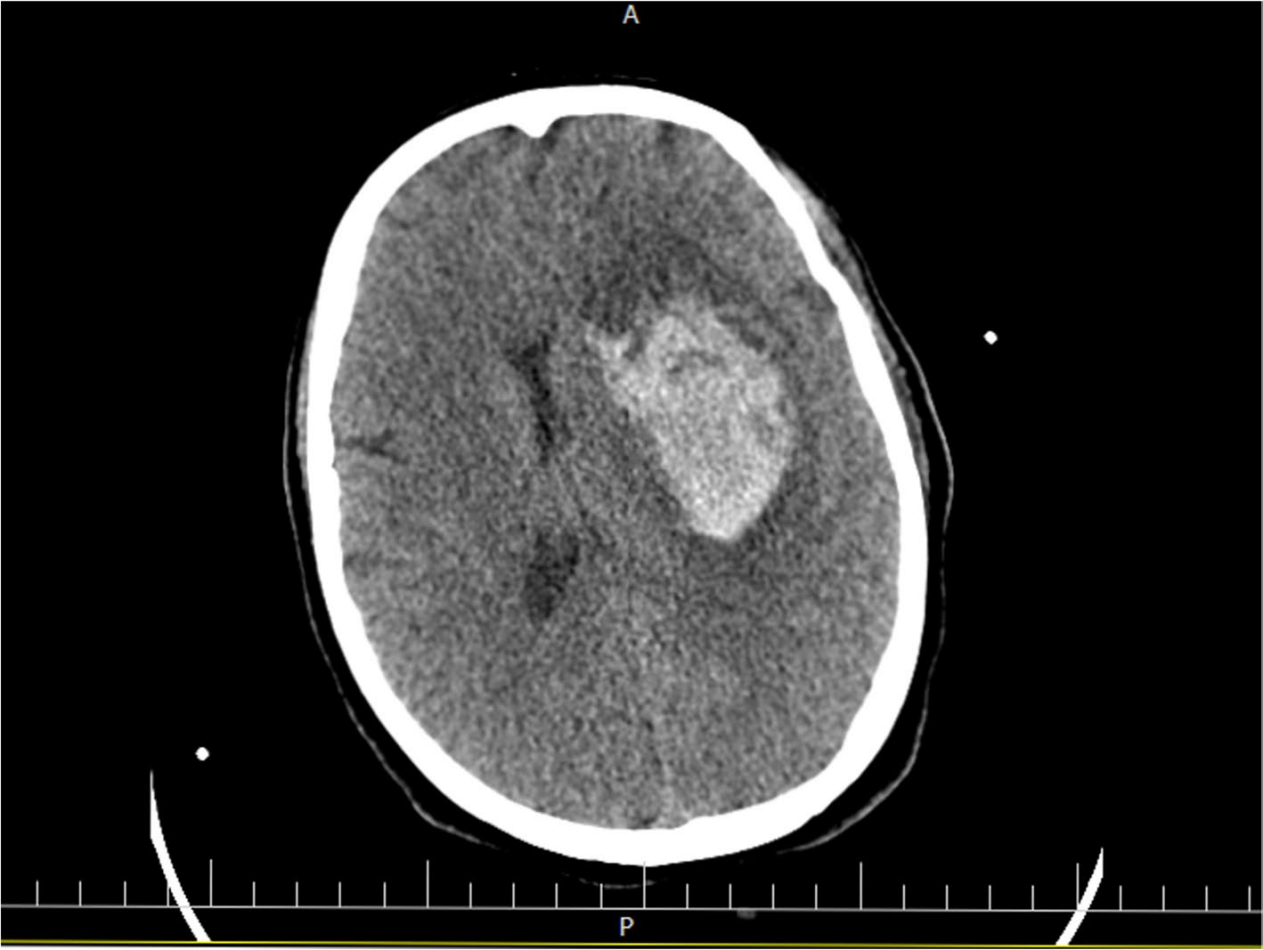
Axial view of brain CT scan showed a 30*56 mm hyperdense mass-like lesion in the left fronto-parietal area, extensive hemorrhage in left basal ganglia and internal capsule, with midline shift and mass effect

**Fig. 2:**
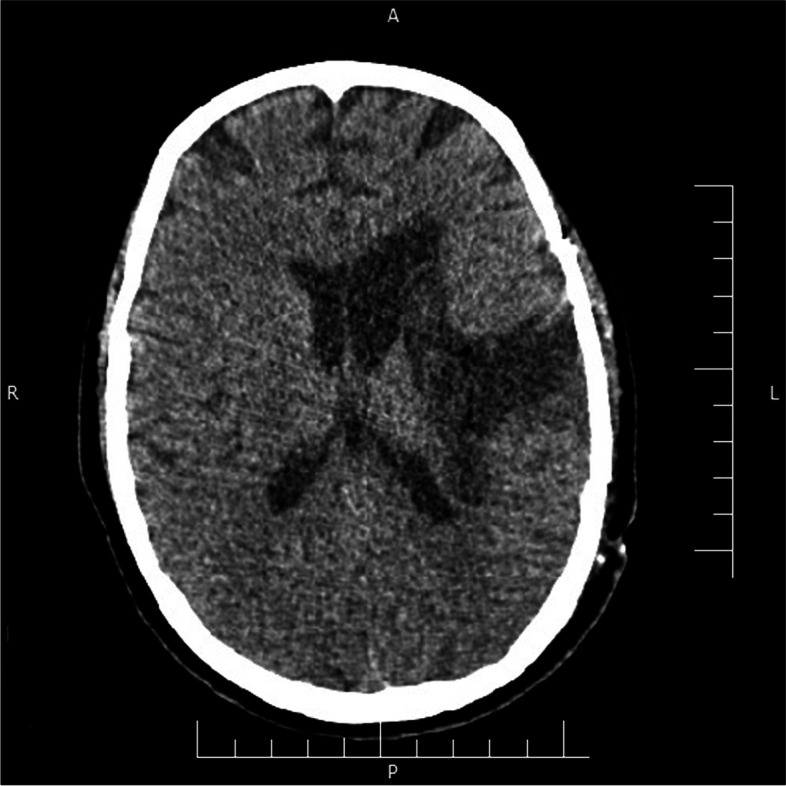
Brain CT scan showed no signs in favor of rebleeding at the one-week follow-up

The patient was a 27 years old woman, Gravid3, Para 3, Live 3 (G3P3L3) with no prior history of any disease. She had a repeat Cesarean delivery a week before the symptom onset. The patient was presented to the emergency department, with decreased level of consciousness, seizure-like movements and a history of headache. She was infected with COVID-19 (confirmed by nasopharyngeal swab test for SARS-CoV-2 PCR), 2 weeks before the onset of neurological symptoms. She did not receive any anti thrombosis prophylaxis followed by cesarean delivery. On admission, her vital signs were as follows: blood pressure 160/90 mmHg, heart rate 88 beat/min, respiratory rate 24 breaths/min with oxygen saturation of 92% on air room and temperature 37.9 °C. Brain CT scan showed a 30*56 mm hyperdense mass-like lesion in the left fronto-parietal area, with midline shift and mass effect (Fig. 1). Her past medical history, was not consist of abnormal coagulation profile before or after delivery. She had normal coagulable laboratory tests during the admission with normal platelet count, as well. Anticonvulsant therapy (Phenytoin) was commenced and the patient was scheduled for an emergent surgery. Given the history of COVID-19 and ground-glass opacities in the chest CT scan, Remdesivir, Interferon, Cefepime, Dexamethasone and Sofosbuvir were commenced for the patient. She was transferred to the operating room and an urgent evacuation of hematoma via a left fronto-temporo-parietal craniotomy approach was performed. Evacuation of hematoma (≈ 200 cc), drain insertion and duraplasty were performed successfully with no postoperative bleeding. After the surgery, patient was admitted to the intensive care unit. She became extubated a day after surgery. Three days later, a 40 °C fever started. Therefore, with diagnosis of Hospital acquired pneumonia (HAP), Colistin and Teicoplanin were ordered at this time. The patient became afebrile, but she was aphasic and had right hemiplegia. However, the neurological symptoms of the patient gradually improved within a week. Follow-up brain CT scan was uneventful and the patient was discharged without any serious complications (Fig. 2). On the 2-month follow-up, the patient reported no significant disability. Meanwhile, she refrained to be evaluated by diagnostic cerebral angiography.

## Discussion and conclusion

Since the declaration of COVID-19 pandemic, hundreds of studies have been published from different countries about the characteristics of patients with COVID-19. Among them, some considerable studies have issued the extra-pulmonary complications of SARS-CoV-2 infection [10]. Almost every part of human body can be affected by these complications. Though, hemostasis balance, vessels and central nervous system might be regarded as the most important ones [11]. Occurrence of different types of intracranial hemorrhage in patients with COVID-19 is a known phenomenon and was systematically reviewed by Cheruiyot et al. According to this study, out of 148 individuals with a diagnosis of different intracranial hemorrhage, extracted from 23 studies, multiple cranial compartments (MCH) was reported in 14 cases (9.5%), intraparenchymal hemorrhage (IPH) was reported in 93 (62.6%), subarachnoid hemorrhage (SAH) in 22 (15.0%), subdural hemorrhage (SDH) in 17 (11.6%), and isolated intraventricular hemorrhage (IVH) were reported in only 2 cases [12]. To the best of our knowledge, occurrence of intracranial hemorrhage in a pregnant woman infected with COVID-19 have not been reported so far.

In addition to the increased risk of spontaneous ICH in patients with COVID-19, pregnancy itself can be considered as a risk factor for nontraumatic ICH. about which, Meeks et al. demonstrated an increased rate of ICH during the third trimester and the first 12 postpartum weeks [13]. Data from previous studies demonstrated an increase in the risk of ICH in pregnant women with eclampsia, gestational hypertension, coagulopathy, and history of tobacco use by increasing the states of cerebral hyper-perfusion [13]. However, we observed none of these factors in this case. Given the above findings, pregnant women with SARS-CoV-2 infections might be at a higher risk for spontaneous ICH. Therefore, physicians must be aware of hemorrhagic complications at the time of COVID-19 pandemic. Especially, in pregnant individuals suspicious of COVID-19 infection.

In conclusion, pregnant women infected with COVID-19 seem to be more prone to develop ICH. Therefore, any symptoms pertaining to the central nervous system must be assessed for possible kinds of intracranial hemorrhage. The concurrence of the post-partum period and COVID-19 may be one of the causes of cerebral hemorrhage in this patient. However, the causation is not certain and suggested to investigate the possibility of causality or coincidence in further studies.

## Data Availability

Not applicable.

## Abbreviations

COVID-19: Coronavirus Disease 2019
SARS-CoV-2: Respiratory syndrome coronavirus 2
ICH: Intracerebral Hemorrhage
WHO: World Health Organization
2019-nCoV: 2019 novel coronavirus
HAP: Hospital acquired pneumonia
MCH: Multiple cranial compartments
IPH: Intraparenchymal hemorrhage
SAH: Subarachnoid hemorrhage
SDH: Subdural hemorrhage
IVH: Intraventricular hemorrhage
